# Supplementary material for: Knockout of the OsNAC006 Transcription Factor Causes Drought and Heat Sensitivity in Rice
Source: Int J Mol Sci. 2020 Mar 26;21(7):2288. doi: 10.3390/ijms21072288 (PMC7177362; doi:10.3390/ijms21072288)
Supplement: Supplementary file 1 [file ijms-21-02288-s001.zip › ijms-744714-publish supplementary/2020-01-20-OsNAC006 supplymental figures, V18.pdf]

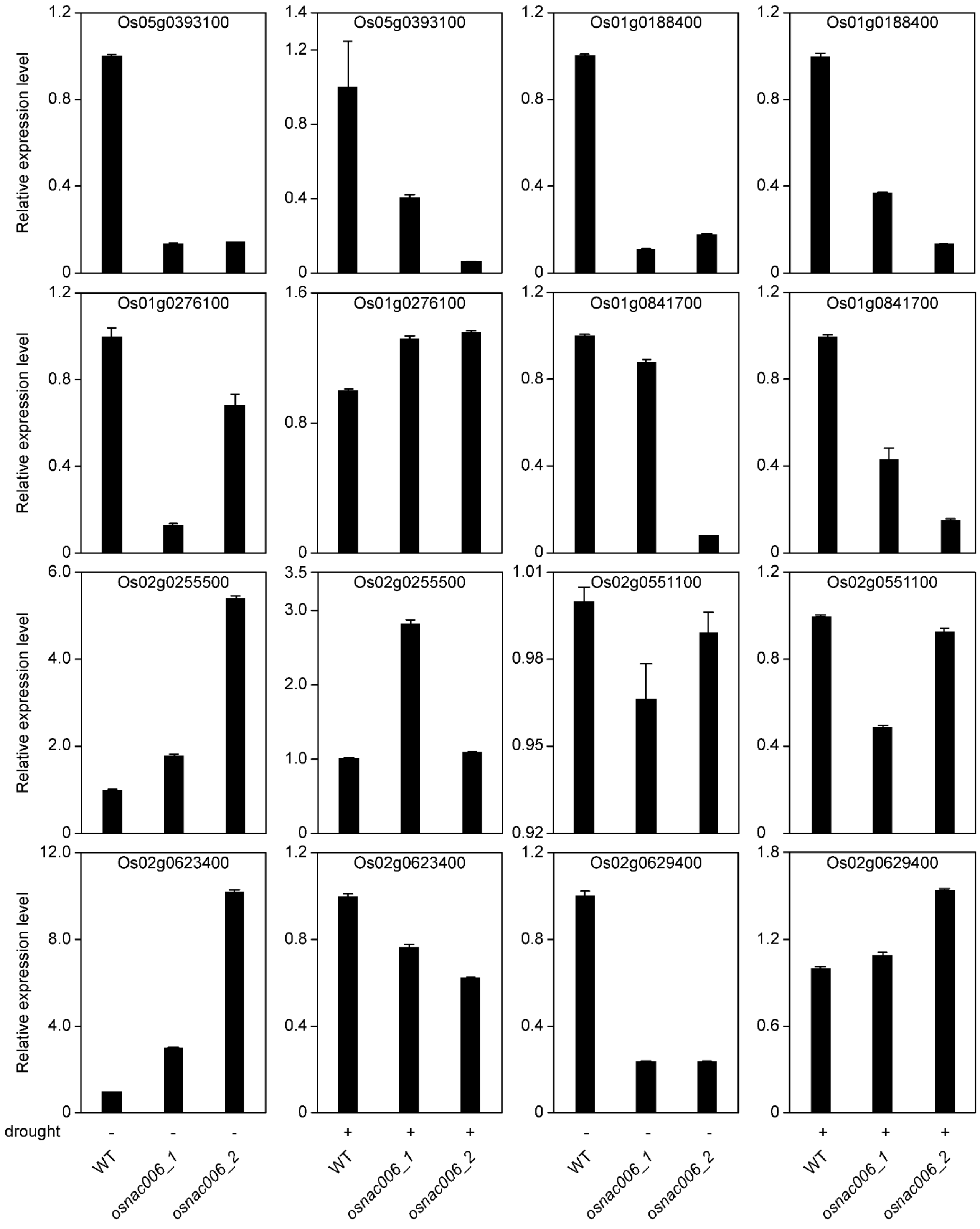

Supply figure1 **Validation of the RNA-seq results by qRT-PCR. Error bars indicate the se based on three technical replicates.**

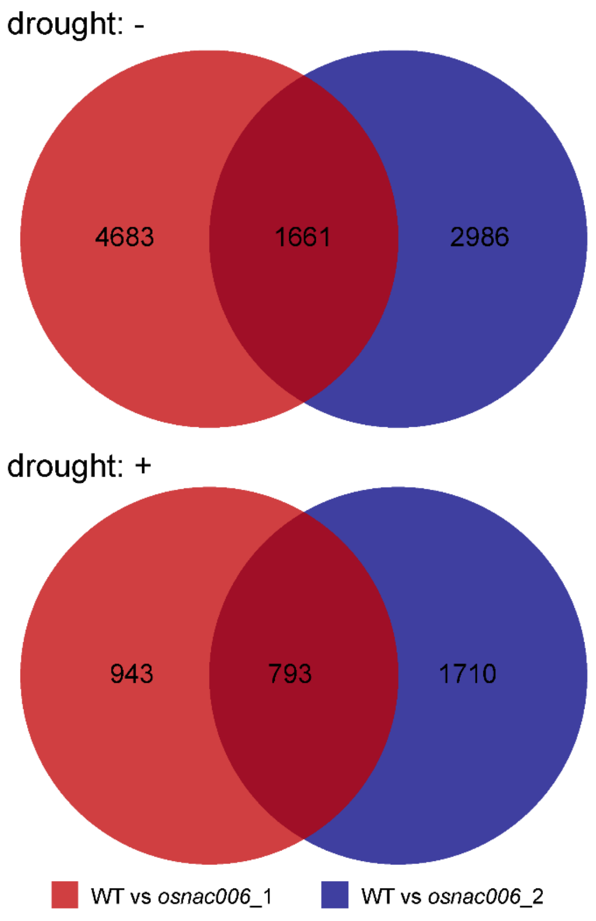

Supply figure2 Venn diagram analysis of the DEGs

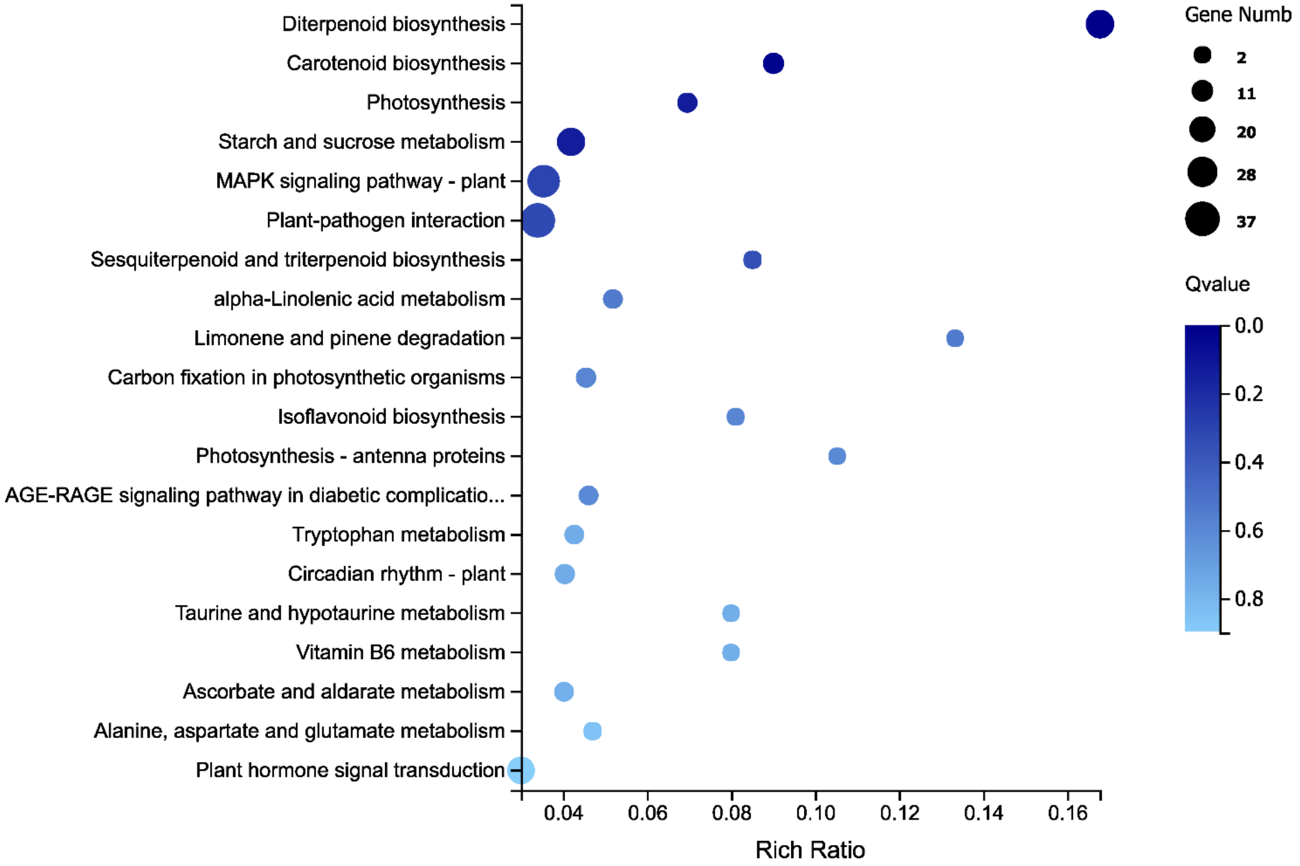

Supply figure3 KEGG analysis of response to stimulas -related DEGs

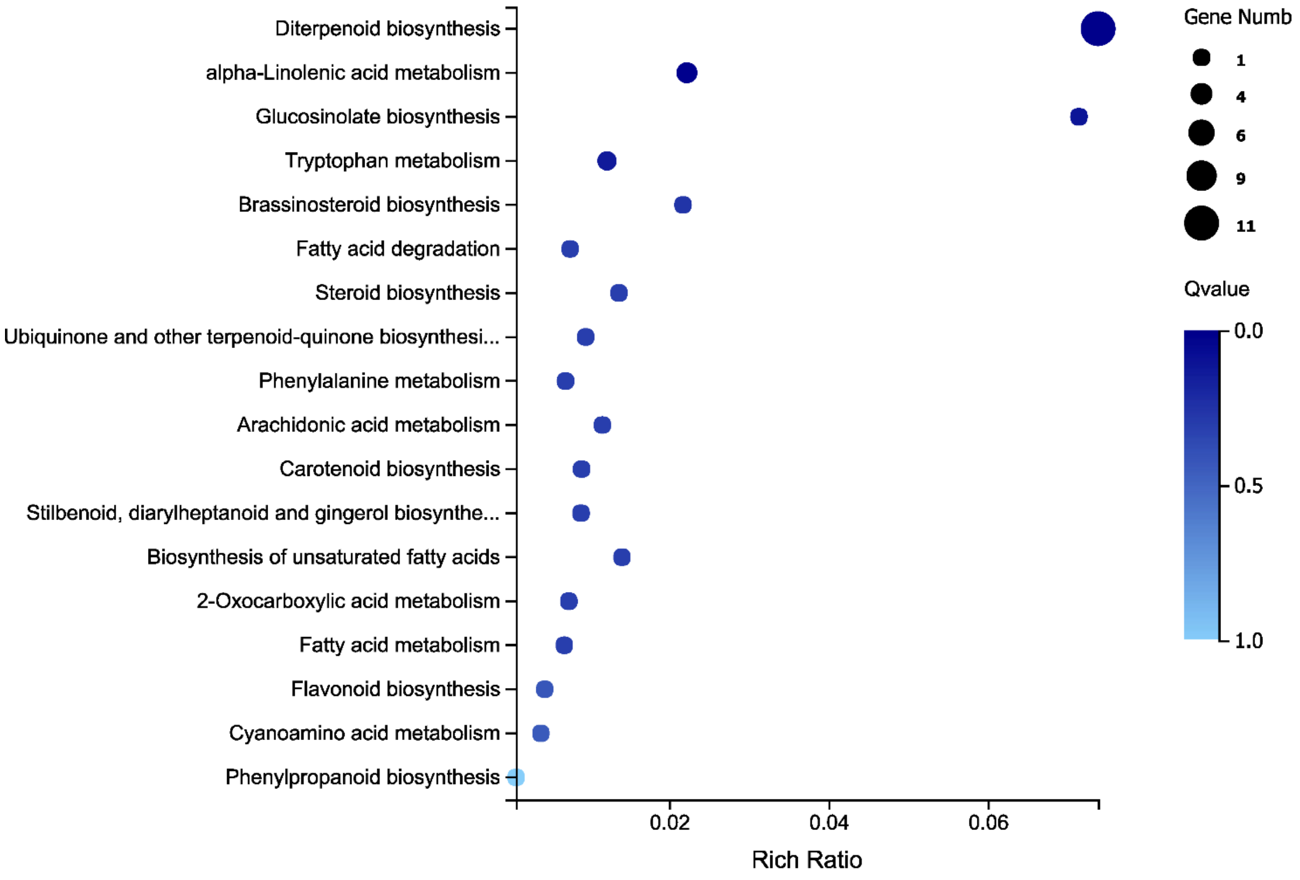

Supply figure4 KEGG analysis of oxidoreductase activity -related DEGs

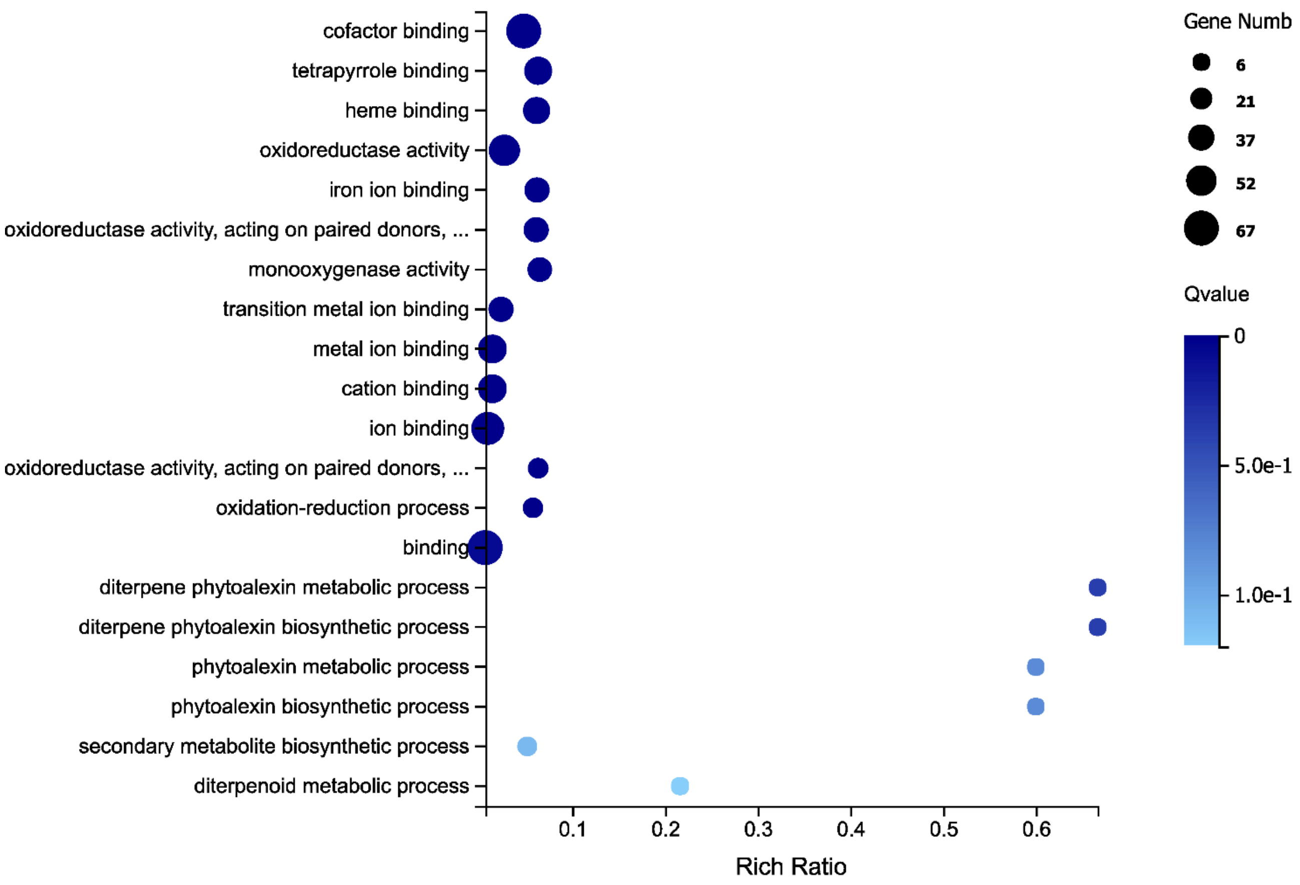

Supply figure5 KEGG analysis of cofactor binding-related DEGs

Supply figure5

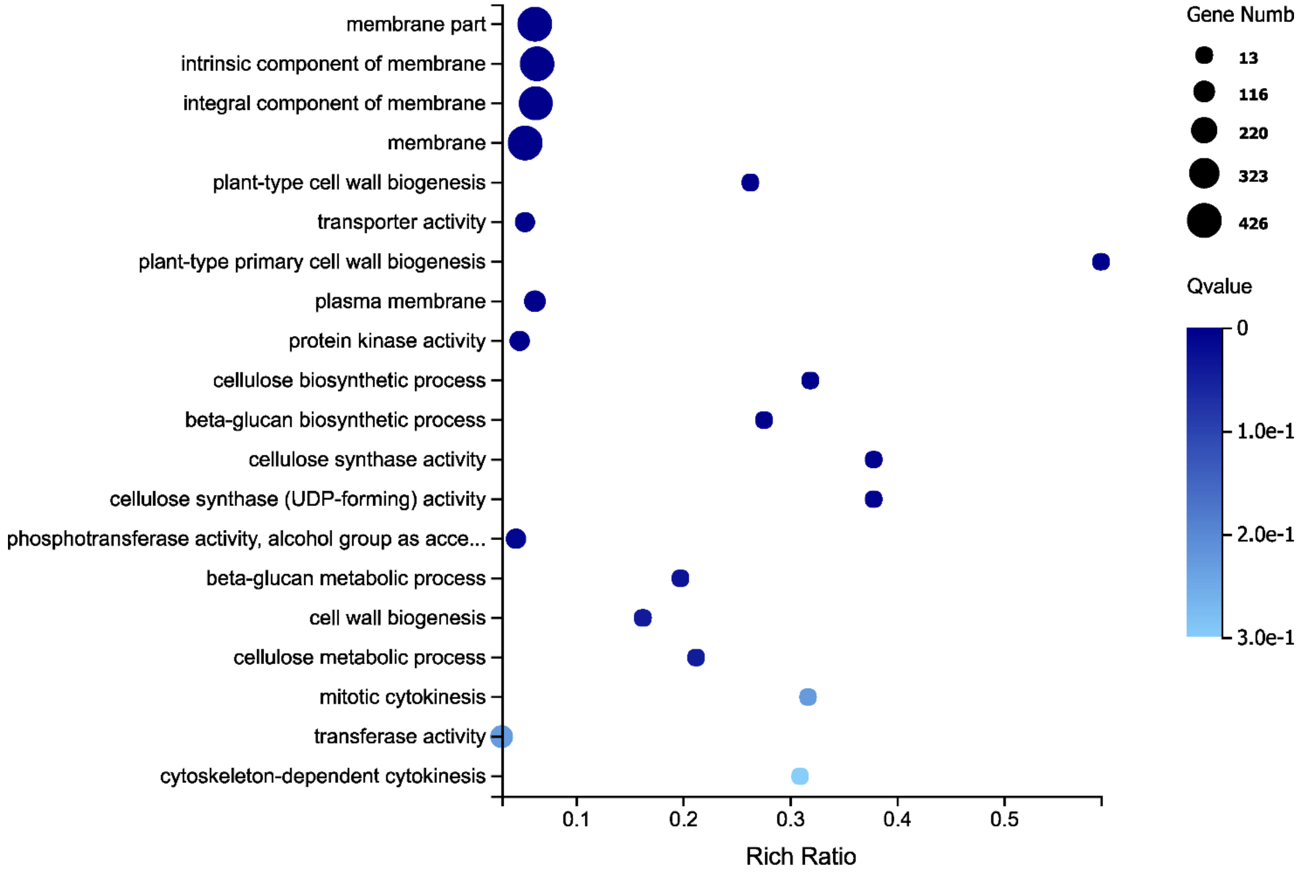

Supply figure6 KEGG analysis Membrance part-related DEGs
