# Supplementary material for: Knockout of the OsNAC006 Transcription Factor Causes Drought and Heat Sensitivity in Rice
Source: Int J Mol Sci. 2020 Mar 26;21(7):2288. doi: 10.3390/ijms21072288 (PMC7177362; doi:10.3390/ijms21072288)
Supplement: Supplementary file 1 [file ijms-21-02288-s001.zip › ijms-744714-publish supplementary/2020-03-25-tables.docx]

**Supplemental Table 1 Oligonucleotides used in this study**

| Primer name | Primer sequence |
| --- | --- |
| OsActin-RT-F | AGCTGCGGGTATCCATGAGA |
| OsActin-RT-R | GCAATGCCAGGGAACATAGTG |
| DCAS9-R | TCTTCTCACCAGGGAGCTGAGCA |
| DCAS9-F | CATATGCAGCAGCTATATGTGGA |
| OsNAC006-cq-F | ATGGTGAGCGGCCGGCAGAA |
| OsNAC006-cq-R | TCACACCTGAAATTTGTCGTGGTGGA |
| OsNAC006-RT-F | CGAGGAGGTTGGTATAAGTGGTGA |
| OsNAC006-RT-R | TATTTGATACAGGGTTCGTCGGC |
| OsNAC006-CX-F | CTGTCAAGAAGCATAGGCCACC |
| OsNAC006-CX-R | TGCTTCAAATGCTGCATCTCG |
| Os01g0846300-RT-1F | ATGGATGGGGAGGTCGGTAA |
| Os01g0846300-RT-1R | CTCGATTCGTTCCAGCTCGT |
| Os01g0846300-RT-2F | GGGGATAAGCAAGGCGGAGT |
| Os01g0846300-RT-2R | CGACGACGACGCTGACATTG |
| Os02g0551100-RT-1F | CGGCACCCAAACATCATCCG |
| Os02g0551100-RT-1R | CAGCTTCAAATCCCGGTGGC |
| Os04g0301500-RT-1F | GCGTCCTGACGAGGATTCGG |
| Os04g0301500-RT-1R | ACGTCGTCGCAACATGGGAG |
| Os05g0393100-RT-1F | GATGCACATGCCCTGGAAGC |
| Os05g0393100-RT-1R | ATCAGTCAGAGCGGTGAGCC |
| Os01g0188400-RT-1F | GGTGGTTGGTGGAACCCTGG |
| Os01g0188400-RT-1R | GCCAAACCTTCTTGCGGCAC |
| Os01g0276100-RT-1F | CCAAGACCAATGGCCGGGAG |
| Os01g0276100-RT-1R | CAGCTGGGTGTCAAGCCCAA |
| Os01g0841700-RT-1F | ACCACGACACCTTCTCACGG |
| Os01g0841700-RT-1R | TCCAACCTGTTCTGCGTGGT |

**Supplemental Table 2 Off-target analysis of *OsNAC006-sgRNA01***

| **Off-target sites** | **No of mismatches** | **Locus** | **Region** | **Confirmed** |
| --- | --- | --- | --- | --- |
| AGATGCTCCCCCGACCACGGGGG | 4 | Chr4: -21622903 | Intergenic | No |
| TGCTGCTCCGCCGGCCGCGGTGG | 4 | Chr11: -4324425 | Intron | No |
| AGGAGGTCGTCCGACAGCGGCGG | 4 | Chr1: -27871126 | UTR | No |
| AGCTGCTCCAGCGACAGCGGCGG | 4 | Chr5: +15918278 | CDS | No |
| The PAM motif (NGG) is shaded gray. Mismatched bases are colored red. | | | | |
